# The Mechanistic Target of Rapamycin Mediates *Clostridium perfringens*-Induced Chicken Necrotic Enteritis Attenuated by Secondary Bile Acid Deoxycholic Acid

**DOI:** 10.3390/microorganisms13040762

**Published:** 2025-03-27

**Authors:** Mohit Bansal, Tahrir Alenezi, Ying Fu, Janashrit Shrestha, Ayidh Almansour, Hong Wang, Anamika Gupta, Rohana Liyanage, Xiaolun Sun

**Affiliations:** 1 Center of Excellence for Poultry Science, University of Arkansas, Fayetteville, AR 72701, USA; fu.ying.work@outlook.com (Y.F.); js206@uark.edu (J.S.); hxw01@uark.edu (H.W.); agupta2@uab.edu (A.G.); 2 Cell and Molecular Biology Program, University of Arkansas, Fayetteville, AR 72701, USA; tjalenez@uark.edu (T.A.); amamansour@sfda.gov.sa (A.A.); 3 College of Medical Applied Sciences, The Northern Border University, Arar 91431, Saudi Arabia; 4 Department of Chemistry, University of Arkansas, Fayetteville, AR 72701, USA; rliyana@uark.edu

**Keywords:** host immune response, bile acid, ileitis, immune cell infiltration, cell death

## Abstract

*Clostridium perfringens* is a prevalent gut bacterial pathogen in humans and animals. This study investigated the role of the mechanistic targets of rapamycin (mTOR) and deoxycholic acid (DCA) on *C. perfringens* intestinal infection. Chickens were sequentially infected with *Eimeria maxima* and received the mTOR inhibitor rapamycin and DCA. *C. perfringens*-induced necrotic enteritis (NE) was evaluated using body weight gain (BWG), histopathology, bile acids, pathogen colonization, cell infiltration and death, and gene expression. The significant difference of *p* < 0.05 was analyzed by one-way ANOVA and multiple comparisons. Notably, rapamycin strongly reduced the subclinical and clinical NE histopathologies. DCA and DCA combined with rapamycin alleviated clinical NE and BWG loss. Rapamycin, DCA, and DCA + rapamycin attenuated bile acid reduction in NE birds, and they also reduced immune cell infiltration into the intestinal lamina propria as well as immune cell migration in vitro. At molecular levels, DCA and DCA + rapamycin reduced proinflammatory *IFNγ*, *MMP9*, *IL23*, and *IL17* gene expression. Rapamycin, DCA, and DCA + rapamycin reduced NE-induced intestinal cell apoptosis. Together, these results suggest that mTOR signaling mediates *C. perfringens*-induced ileitis, and combining mTOR inhibition and DCA improves the intervention efficacy against NE ileitis and BWG loss.

## 1. Introduction

*Clostridium perfringens* is a prevalent gut bacterial pathogen that possesses more than 20 virulent toxins and enzymes [1]. The intestinal loop model has long been documented in investigating toxin virulence in the biomedical field [2]. Although chicken necrotic enteritis (NE) has been induced by manipulating diet composition with high animal protein and high fiber, etc., NE in poultry farm settings mainly results from coccidiosis and *C. perfringens* infection at 2–6 weeks of age [3], causing an annual loss of USD 6 billion in the poultry industry [4]. During clinical NE, birds suffer severe diarrhea and mortality, anorexia, and reduced BWG and feed conversion efficiency [5]. Most NE incidences are subclinical, and the signs of enteritis are subtle and difficult to differentiate between healthy and NE birds. Nevertheless, NE could be a valuable animal model of natural infection to study *C. perfringens* gut infection pathogenesis.

Despite limited knowledge of its molecular and cellular events, NE induces ileitis and inflammatory signaling pathways. Various adaptive and innate immunity actively mediate intestinal inflammation, such as the T helper (Th) cell types Th1, Th2, regulatory T (Treg), Toll-like receptor (TLR), phosphatidylinositol 3-kinases (PI3K), mTOR, cyclooxygenase (COX), and nuclear factor-κB (NF-κB) [6,7]. PI3Kγ signaling mediates neutrophil migration into colonic tissues and is essential for *Campylobacter jejuni*-induced intestinal inflammation [8]. mTOR, a downstream target of PI3K, modulates a number of essential functions, including cell growth, proliferation, survival, and innate and adaptive immune responses [9,10,11]. The mTOR inhibitor rapamycin reduced *C. jejuni*-induced campylobacteriosis enteritis in *Il10^−/−^* mice [12]. The signaling pathways induce the downstream expression of various proinflammatory cytokine and chemokine proteins, such as IFNγ, IL17a, IL1β, IL8, and TNFα [13]. Inflammatory cytokines and chemokines mediate immune cell migration to inflamed sites, amplify inflammatory response, and cause cell death [14]. Cell death is categorized into accidental cell death and regulated cell death (RCD), and some RCDs induce apoptosis, necrosis, necroptosis, and pyroptosis [15]. NE induces gene expression of proinflammatory cytokines such as *IFNγ* and *IL1β* in chicken intestinal tissue [16,17,18]. The COX pharmacological inhibitor aspirin reduces clinical NE ileitis [16]. Notably, apoptosis was induced in the ileum of NE birds [16].

Bile acids have been extensively investigated for their essential role in lipid digestion and absorption [19]. Bile acids synthesized in the liver are secreted into the intestine and metabolized by the gut microbiota [20]. Bile acids are composed of primary bile acids in the conjugated form of tauro- or glyco-cholic acids (T-CA or G-CA) and chenodeoxycholic acids (T-CDCA or G-CDCA), as well as deconjugated CA and CDCA [21]. Primary bile acids are metabolized by intestinal microbiota into secondary bile acids such as deoxycholic acid (DCA), lithocholic acid (LCA), and ursodeoxycholic acid UDCA [20]. Accumulating evidence shows that bile acids play a key role in infectious diseases, including *C. difficile* [22], NE [16], and campylobacteriosis [23].

In this study, we aimed to investigate the cellular and molecular mechanisms of *C. perfringens* infection using chicken NE, the mTOR inhibitor rapamycin, DCA, and a cell culture system. The findings from this study will be important for a better understanding of *C. perfringens* infection and for developing effective interventions.

## 2. Materials and Methods

### 2.1. Chicken Experiments

The chicken experiments were performed at the Poultry Health Laboratory of the University of Arkansas Fayetteville, Arkansas. Animal experiments were conducted in accordance with Animal Research: Reporting of In Vivo Experiments (https://www.nc3rs.org.uk/arrive-guidelines, accessed on 12 October 2018). All the experiments were approved by the Animal Care and Use Committee of the University of Arkansas. Day-old meat-type chicks (broilers) obtained from Cobb-Vantress (Siloam Springs, AR, USA) were wing-tagged and randomly allocated to individual floor or cage pens. The birds were raised on their respective diets and water ad libitum. The temperature was maintained at 34 °C during the first five days and gradually lowered to 23 °C on day 25 or 26. Corn-soybean meal-based starter diets and grower diets were used during days 0–10 and 11–26. The basal diet was formulated to meet the nutritional requirements for broiler chickens based on the National Research Council [24] and the breeder’s recommendations [25]. Antibiotics, coccidiostats, or enzymes were not added to the feed. On day 18, the birds were orally gavaged with ~20,000 sporulated *E. maxima* M6 oocysts, which were kindly provided by Dr. Hargis’ lab, and on days 23 and 24, they were orally infected with 10^9^ CFU *C. perfringens*/bird, as described before [16]. This *C. perfringens* isolate was used in our previous studies [16,26,27].

In the subclinical NE experiment of floor pens, the noninfected, NE, and NE + Rap-1 groups had 14, 16, and 14 birds (1 pen/group) during days 0–23 as well as 8, 10, and 11 birds (1 pen/group) during days 0–23 and 23–26, respectively. The NE + Rap-1 birds were subcutaneously injected with 1500 μg/kg body weight rapamycin (LC Laboratories, Woburn, MA, USA) from days 17 to 25. Subclinical NE was unexpected and resulted from weakened *E. maxima*, which was later found due to long-term storage.

In the clinical NE experiment of cage pens, the birds were fed rapamycin 75 (R 75) and 300 μg/kg feed (R 300), 1.5 g/kg feed DCA (Alfa Aesar, Stoughton, MA, USA), or DCA + R 300 for days 14–25. The noninfected, NE, NE + R 75, NE + R 300, DCA, and DCA + R 300 groups had 9 (2 pens), 11 (2 pens), 7 (1 pen), 15 (2 pens), 14 (2 pens), and 14 (2 pens) birds during days 0–23, as well as 6 (2 pens), 7 (2 pens), 5 (1 pen), 10 (2 pens), 10 (2 pens), and 10 (2 pens) birds during days 23–25, respectively. Only the NE group had 2 dead birds (not included in the final 7 birds) during days 24–25.

The chicken body weight was individually measured on days 0, 14, 18, 23, and 25 (the clinical trial) or 26 (the subclinical trial). Two to six birds were randomly selected and euthanized on day 23 to lower the bird density, and the intestine was collected for hematoxylin and eosin (H&E) staining (no result was reported in this report). The rest of the birds were euthanized on day 25 (the clinical trial) or 26 (the subclinical trial) to collect ileal tissue and digesta so as to analyze the histopathology, pathogen colonization, inflammation markers, and bile acids.

### 2.2. Histopathology and Quantification of Ileal Immune Cell Infiltration

Chicken NE ileitis was assessed using a microscopic histopathological evaluation of the Swiss-rolling small intestine around Meckel’s diverticulum (approximately 8 cm down), as described before [28,29]. Briefly, the Swiss-rolled ileal tissue samples were fixed in formalin (pH 7.4) overnight at 4 °C, embedded in paraffin, cut into 5 μm tissue sections, and processed and stained with H&E at the Histology Laboratory in the Department of Poultry Science at the University of Arkansas at Fayetteville. Images were acquired using a Nikon TS2 fluorescent microscope (Nikon Inc., Melville, NY, USA). The NE ileal histopathology score was evaluated for inflammation based on characteristic NE lesions, as described before [26]. The total histopathological scores were calculated by adding the four randomly selected area scores on a scale of 0–4.

To quantify the immune infiltration into ileal lamina propria, briefly, the ileal tissue slides were deparaffinized three times with a xylene wash and then rehydrated with 100%, 95%, and 70% ethanol. To visualize the cell nuclei, the slides were stained with DAPI. Using the Nikon TS2 fluorescent microscope, the fluorescent blue cell nuclei were imaged and enumerated using Fiji ImageJ (1.54f). The number of infiltrated cells was calculated relative to that of the noninfected bird cells.

### 2.3. Quantification of Ileal Bile Acids Using Targeted Metabolomics

The ileal digesta was collected, and the bile acid composition was analyzed using multiple reaction monitoring (MRM) mass spectrometry at the Statewide Mass Spectrometry Facility at the University of Arkansas in Fayetteville, Arkansas, as described before [28]. Briefly, the ileal digesta samples were extracted for bile acids using the methanol method. To identify and quantify the bile acids, MRM methods have been developed for the nine bile acids isotopically labeled (some with deuterium and some with five deuterium) and unlabeled standards, as described before [26]. Calibration curves with internal standards were used to estimate the bile acid concentration. Liquid chromatography–tandem mass spectrometry (LC-MS/MS) analysis was performed in the negative-ion mode using a Shimadzu UPLC-20A/LC-30A coupled with a Shimadzu 8050 triple quadrupole mass spectrometer (Shimadzu Scientific Instruments, Columbia, MD, USA). LC separation was performed using a ZORBAX RRHD Eclipse Plus C18 Column with a 2.1 × 150 mm, 1.8 µm, 1200 bar C18 column (Agilent, Santa Clara, CA, USA) at a flow rate of 0.3 mL/min. The three most intense multiple reaction monitoring (MRM) fragments for each bile acid standard were used, as described before [26].

### 2.4. C. perfringens and E. maxima Colonization

The ileal digesta samples from birds at day 25 were collected and weighed, and DNA was extracted using the phenol/chloroform method, as previously described [26]. Real-time PCR was performed to quantify the colonization level of *C. perfringens* and *E. maxima* in the ileal digesta by targeting the *16S* and *18S* rDNA, respectively. *C. perfringens* *16S* rDNA was detected using the following primer sets: *16S* forward 5′-AGGAGCAATCCGCTATGAGA-3′; *16S* reverse 5′-GTGCAATATTCCCCACTGCT-3′ and *E. maxima* (*Em*) *18S* forward 5′-GACCTCGGTCACCGTATCAC-3′; *E. maxima* (*Em*) *18S* reverse 5′-CGTGCAGCCCAGAACATCTA-3′.

### 2.5. Immune Cells Migrations Assay

Blood was collected from two 5–8 weeks of age BL6 mice, and red blood cells were lysed in a buffer containing 8.3 g/L of NH_4_Cl in 0.01 M Tris–HCl buffer at pH 7.5. The collected white blood cells (WBCs) were resuspended in a 1% FBS RPMI 1640 medium. *C. perfringens* was inoculated at 10^7^ CFU/well in the bottom wells of 24-well plates except for the negative controls. The medium was triplicated with 100 nM rapamycin, 100 μM DCA, or DCA + rapamycin. The WBCs were plated at 10^4^ cells per insert in Transwells (Corning, Corning, NY, USA) with 3 μm pores and were incubated at 37 °C and 5% CO_2_ for 3.5 h. White blood cells that migrated into the bottom well were imaged and counted one hour later using the Nikon TS2 Microscope system, as described previously [30].

### 2.6. Host Inflammatory Gene Expression and Cellular Apoptosis Using Terminal Deoxynucleotidyl Transferase dUTP Nick End Labeling (TUNEL) Assay

The TRIzol-extracted total RNA was from the ileal tissue samples of birds at d 25 as described before [16,26], and the cDNA was prepared using the Moloney murine leukemia virus reverse transcriptase (M-MLV) (NE Biolabs, Ipswich, MA, USA). The accumulation of proinflammatory genes *IFNγ*, *MMP9*, and *GAPDH* in ileum tissue was determined using the SYBR Green PCR Master mix (Bio-Rad, Hercules, CA, USA) on a Bio-Rad 384-well Real-Time PCR System, as described before [16]. The primer sequences for each gene were as follows: *MMP9* forward: 5′-CCAAGATGTGCTCACCAAGA-3′; *MMP9* reverse: 5′-CCAATGCCCAACTTCTCAAT-3′; *INFγ* forward: 5′-AGCCGCACATCAAACACATA-3′; *INFγ* reverse: 5′-TCCTTTTGAAACTCGGAGGA-3′; *IL22* forward: 5′-CTGCCCATAGCTGCAGTACA-3′; *IL22* reverse: 5′-CAGTGAAGTGGAGCCACAAA-3′; *IL23* forward: 5′-ATGCATTGCGATGTCTGAAG-3′; *IL23* reverse: 5′-ACTTGGGTGCTTCCAAGATG-3′. The gene expression of the fold change was calculated using the ΔΔCt method [16,26] and *GAPDH* as an internal control [16].

The degree of ileal cell apoptosis in the intestinal tissue was assessed using the TUNEL assay, as described before [16,26]. The TUNEL assay detects the late phase of cellular apoptosis based on the fragmentation of nucleic acids. In brief, ileal tissue slides were deparaffinized three times with a xylene wash and then rehydrated with 100%, 95%, and 70% ethanol. The tissues were then incubated at 37 °C for 90 min with the TUNEL solution (5 μM Fluorescein-12-dUTP (Enzo Life Sciences, Farmingdale, NY, USA), 10 μM dATP, 1 mM Tris-HCl pH 7.6, 0.1 mM EDTA, and 1U TdT enzyme (Promega, Madison, WI, USA)). For visualization of the nuclei, the slides were counter-stained with DAPI. Using a Nikon TS2 fluorescent microscope, the fluorescent green apoptotic cells were examined and imaged.

### 2.7. Statistical Analysis

Differences between the treatments were analyzed using a parametric one-way ANOVA followed by multiple comparisons and Dunnett’s correction using Prism software (version 7.0). In the subclinical experiment, the multiple comparisons were NE vs. Noninfected or NE + Rap-1. In the clinical experiment, the multiple comparations were NE vs. Noninfected, NE + R 75, NE + R 300, NE + DCA, or NE + DCA + R 300, as well as NE + DCA + R 300 vs. NE + R 300 or NE + DCA. The values are shown as the means of the samples in the treatment ± standard error of the mean. Experiments were considered statistically significant if the *p*-values were <0.05.

## 3. Results

### 3.1. mTOR Pharmacological Inhibitor Rapamycin Reduced Subclinical NE-Induced Ileitis but Not Body Weight Gain Reduction

**Figure 1:**
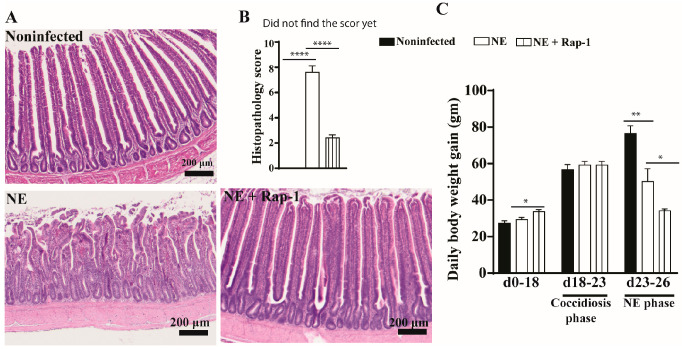
mTOR inhibitor rapamycin prevented subclinical NE-induced ileitis. Cohorts of meat-type chickens (broilers) were subcutaneously injected with 1.5 mg/kg of rapamycin starting from day 17. Birds were challenged with *E. maxima* on day 18 and *C. perfringens* on days 23 and 24. Noninfected, NE, and NE + Rap-1 groups had 14, 16, and 14 birds during days 0–23, as well as 8, 10, and 11 birds during days 0–23 and 23–26, respectively. (**A**) Representative images of H&E-stained ileal tissue on day 26. (**B**) Histopathology score of ileal tissue on day 26. (**C**) Periodic body weight gain (BWG) during days 0–18, 18–23, and 23–26. All graphs show the mean ± SEM. The scale bar is 200 μm. * *p* < 0.05, ** *p* < 0.01, and **** *p* < 0.0001.

To investigate the role of host mTOR signaling in NE, the pharmacological inhibitor rapamycin was used during days 17–25. NE birds showed watery diarrhea (wetter litter) and ruffled feathers. At the cellular level, the intestines of noninfected birds showed evenly distributed long villi and short crypts, whereas the villi of NE birds were notably swollen and shortened, and the crypts were elongated (Figure 1A). The mTOR inhibitor rapamycin significantly alleviated NE-induced ileitis, showing a villus arrangement, long villi, and short crypts with attenuated histopathological scores of 2.4 vs. 7.6 for the NE birds (Figure 1B). Chicken BWG is an important parameter for meat chicken production in the poultry industry, and we measured it. Compared to noninfected birds, NE reduced the chicken body weight gain (BWG) by 33% (50.2 vs. 76.6 g/d/bird), but NE birds still gained body weight. Interestingly, rapamycin reduced BWG by 51% (34.1 vs. 50.2 g/d/bird) despite a significant reduction in NE histopathology (Figure 1C). Based on the NE signs, histopathology severity, and BWG, subclinical NE was determined. These findings indicate that the mTOR signaling pathway mediates subclinical NE-induced ileitis.

### 3.2. Combining Rapamycin and DCA on Reducing Clinical NE

**Figure 2:**
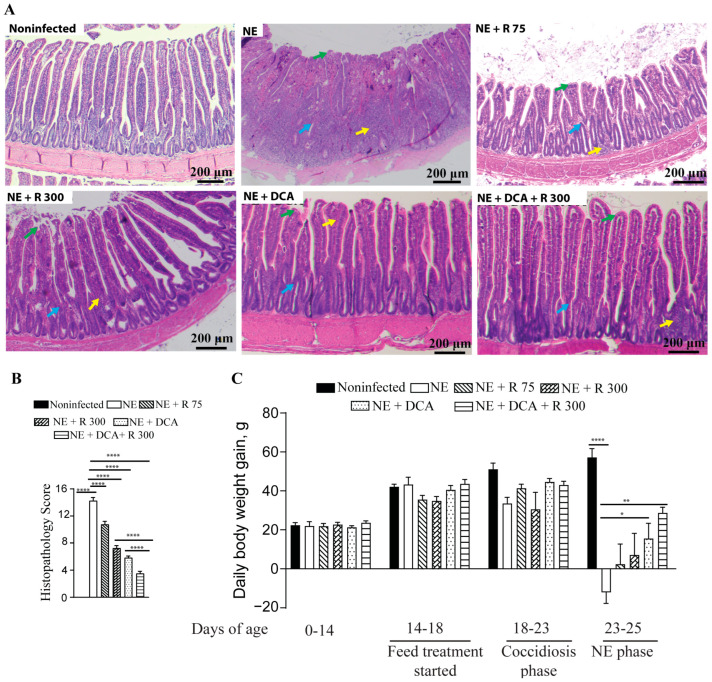
Rapamycin and DCA prevented clinical NE-induced ileitis. Cohorts of broiler chickens were fed rapamycin (75 (R 75) or 300 (R 300) μg/kg feed), DCA (1.5 g/kg feed), and R 300 + DCA starting from 14 days of age. Birds were challenged with *E. maxima* at d 18 and *C. perfringens* at days 23 and 24. Noninfected, NE, NE + R 75, NE + R 300, DCA, and DCA + R 300 groups had 9, 11, 7, 15, 14, and 14 birds during days 0–23, as well as 6, 7, 5, 10, 10, and 10 birds during days 23–25, respectively. (**A**) Representative images of H&E-stained ileal tissue sections showing immune cell infiltration (yellow arrow), cryptic hyperplasia (blue arrow), and blunted villi (green arrow) on day 25. (**B**) Histopathology score of ileal tissue on day 25. (**C**) Bird BWG was measured on days 0, 14, 18, 23, and 25. Periodic BWG at days of 0–14, 14–18, 18–23, and 23–25. All graphs show the mean ± SEM. The scale bar is 200 μm. * *p* < 0.05, ** *p* < 0.01, and **** *p* < 0.0001.

To address the concern of undesirable BWG reduction in the rapamycin group, the combination of DCA and rapamycin was used, and the agents were added to the diet for convenient chicken management. Both DCA and rapamycin were added to the feed for convenient chicken management. During the NE phase, the NE birds showed watery and dark brown diarrhea, ruffled feathers, morbidity, reduced feed consumption (far less feed added), and death. During necropsy, the small intestine with a thin wall was ballooned and filled with stinky gas and watery digesta. As shown in Figure 2A, NE-induced severe ileitis histopathology shows extensively destroyed and fused villi (green arrow), crypts (blue arrow), and massive immune cell infiltration (yellow arrow). Notably, the rapamycin 75 μg/kg feed (R 75), R 300, DCA, and DCA + R 300 relieved the NE histopathology (scores 10.8, 7.3, 5.8, and 3.5 vs. 14.3, respectively), with the last group having the best effect (Figure 2A,B). Upon examining growth performance, during the NE phase of days 23–25, the NE birds lost BWG (−12.0 g/bird/day), whereas the R 75, R 300, DCA, and DCA + R 300 birds gained BWG (2.4, 7.1, 15.5, and 28.7 g/bird/day). Notably, only the DCA and DCA + R 300 birds had significant BWG compared to the NE birds. Based on the NE signs, histopathology severity, and body weight loss, clinical NE was determined. These findings indicate that the mTOR signaling pathway mediates clinical NE-induced ileitis, and DCA attenuates clinical NE ileitis and BWG loss. For the following analysis, we focused on R300 without R 75.

### 3.3. Rapamycin and DCA Attenuated NE-Induced Total Bile Acids Reduction

**Figure 3:**
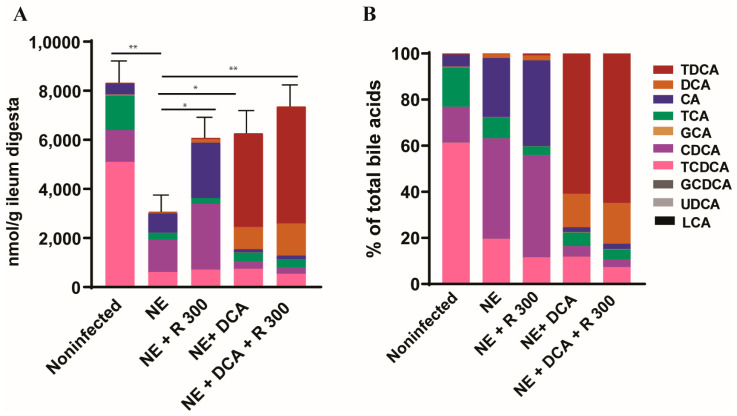
DCA and rapamycin attenuated NE-induced ileal bile acid pool reduction. Cohorts of broiler chicks were fed basal and rapamycin, DCA, or DCA + rapamycin supplemented diets and infected, as seen in Figure 2. Data were analyzed from the noninfected NE, NE + R 300, DCA, and DCA + R 300 groups (6, 7, 10, 10, and 10 birds, respectively). (**A**). Total bile acids quantification in ileum digesta of different groups on day 25. (**B**). Relative composition of bile acids in the ileum digesta of different groups on day 25. All graphs show the mean ± SEM. * *p* < 0.05 and ** *p* < 0.01.

To monitor the changes in the bile acid pool in the experimental birds, we used targeted metabolomics. Notably, clinical NE significantly reduced the total bile acids in the ileum digesta of the NE birds to 36.7% compared to the noninfected birds (3050 vs. 8314 nmol/g digesta, Figure 3A), whereas R 300, DCA, and DCA + R300 restored the total bile acid levels to 72.9, 75.2, and 79.5%, respectively, compared to the noninfected birds. In addition, NE significantly reduced the TCDCA (61.2 vs. 19.5%) but increased the CDCA proportion (15.4 vs. 43.5%) compared to the noninfected birds, possibly resulting from *C. perfringens* bile salt hydrolase deconjugation activity. Interestingly, R 300 did not change the CDCA proportion compared to the NE birds (44.3 vs. 43.5%), whereas DCA and DCA + R 300 significantly increased the TDCA level in the bile acid pool to 60.9 and 64.9%, respectively, compared to 0.6% in the NE birds (Figure 3B).

### 3.4. DCA Reduced C. perfringens and E. maxima Colonization in Ileum

**Figure 4:**
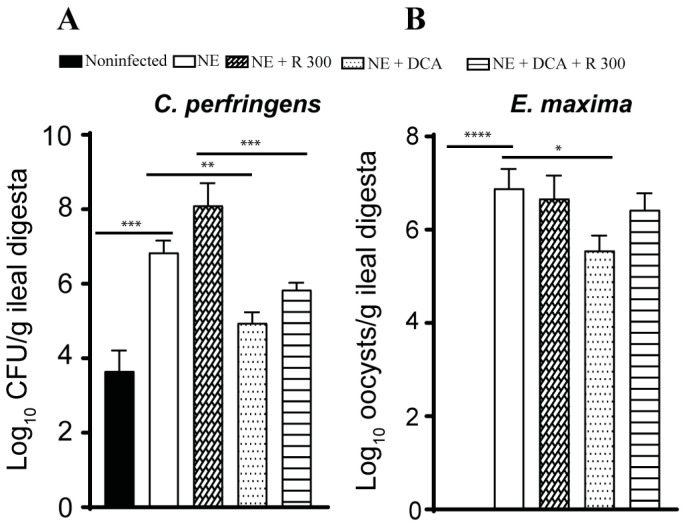
DCA reduced *C. perfringens* and *E. maxima* ileal colonization. Cohorts of broiler chickens were fed rapamycin, DCA, and rapamycin + DCA and infected, as seen in Figure 3. (**A**). *C. perfringens* was quantified in the ileal digesta using PCR on day 25. (**B**). *E. maxima* colonization was quantified in the ileal digesta using PCR on day 25. All graphs show the mean ± SEM. * *p* < 0.05, ** *p* < 0.01, *** *p* < 0.001, and **** *p* < 0.0001.

Pathogen colonization is an important indicator of NE. Because of the lack of a selective culture medium to identify NE pathogens in the digesta, we used real-time PCR targeting *C. perfringens 16S* rDNA and E. maxima *18S* rDNA. NE increased *C. perfringens* colonization by 3.2 log10 CFU/g ileal digesta compared to the noninfected birds, while only DCA alone reduced pathogen colonization by 1.9 log10 CFU/g ileal digesta compared to the NE birds (Figure 4A). Interestingly, R 300 increased *C. perfringens* colonization by 1.3 log10 CFU/g compared to the NE birds. E. maxima colonized in the ileum at 6.9 log10 CFU/g ileal digesta compared to that in the noninfected birds (Figure 4B), suggesting the successful biosecurity measurement of preventing cross-contamination between chicken pens. Interestingly, only DCA significantly reduced E. maxima colonization by 1.4 CFU/g ileal digesta compared to the NE birds.

### 3.5. Rapamycin and DCA Reduced Inflammatory Response

**Figure 5:**
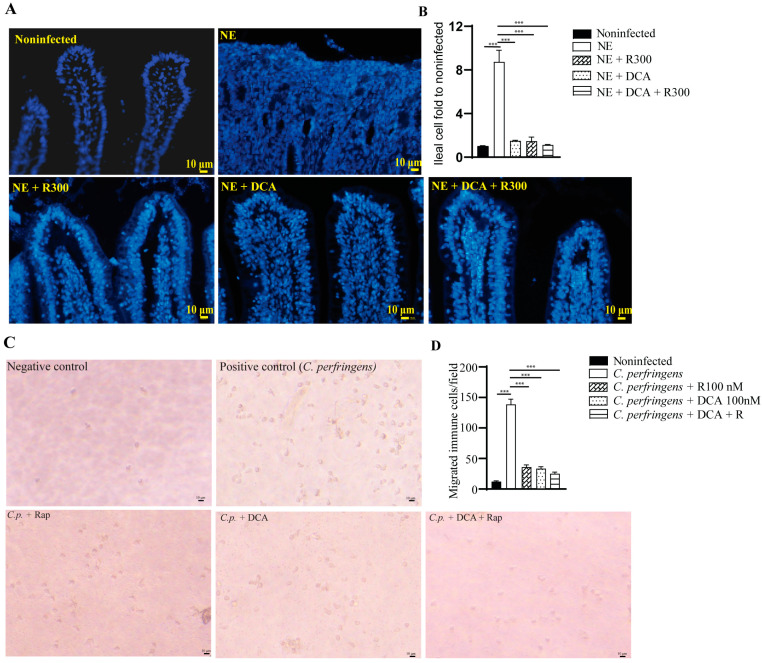
Rapamycin and DCA treatment reduced NE-induced immune cells in vivo and in vitro. Cohorts of broiler chickens were fed DCA, rapamycin, and DCA + rapamycin and infected, as seen in Figure 3. (**A**) Representative images of the DAPI stained ileal tissue sections showing immune cell infiltration on day 25. Data were analyzed from the noninfected NE, NE + R 300, DCA, and DCA + R 300 groups (6, 7, 10, 10, and 10 birds, respectively). (**B**) Immune cell infiltration fold change compared to the noninfected birds on day 25. (**C**) Representative images of murine immune cells migration in response to *C. perfringens*. (**D**) Quantitative measurements of migrated immune cells. All graphs show the mean ± SEM. *** *p* < 0.001.

A notable feature of NE-induced ileitis is the massive infiltration of immune cells into the lamina propria. We then quantified the total number of cells in the ileum using DAPI staining. NE induced strong inflammation and massive immune cell infiltration, and it was difficult to identify the epithelial cell lines (Figure 5A), while other groups had clear epithelial lines. Therefore, we quantified the total number of cells in an image. Notably, NE induced greater than 8.7 folds of the total cells compared to the noninfected birds (Figure 5B). Whereas R 300, DCA, and DCA + R 300 reduced the total immune cells in the ileum by 82.1, 83.5, and 86.7%, respectively, compared to the NE group.

To evaluate whether mTOR signaling mediates immune cell migration [31], we isolated white blood cells (WBCs) from BL6 mice. The WBCs were allocated to the upper inserts, and *C. perfringens* was inoculated in the bottom wells. Compared to the negative control, the positive control of *C. perfringens* induced WBC migration into the bottom wells by more than 11.3 folds (Figure 5C), and the effect was inhibited by rapamycin, DCA, and DCA + rapamycin by 76.7, 75.1, and 82.0%, respectively, compared to the *C. perfringens* group (Figure 5D).

### 3.6. Rapamycin and DCA Reduced Inflammatory Response and Cell Apoptosis

**Figure 6:**
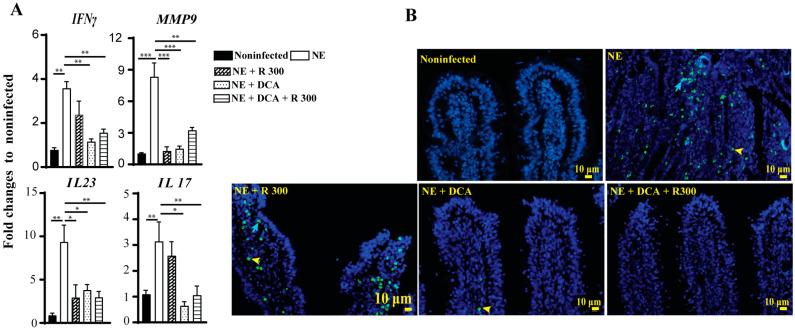
Rapamycin and DCA reduced NE-induced host inflammatory response and cell death. Cohorts of broiler chickens were fed rapamycin, DCA, and rapamycin + DCA and infected, as seen in Figure 3. (**A**) Ileal *IFNγ*, *MMP9*, *IL23*, and *IL17* mRNA fold changes relative to noninfected birds and normalized to *GAPDH* on day 25. (**B**) Representative TUNNEL assay images showing epithelial (blue arrow) and immune cell apoptosis (yellow arrowhead) on day 25. The scale bar is 10 μm. All graphs show the mean ± SEM. * *p* < 0.05, ** *p* < 0.01, and *** *p* < 0.001.

The infiltration of immune cells into the ileum could result from an inflammatory response induced by NE. To investigate this possibility, we assessed the expression of chicken inflammatory mediator mRNA in the ileal tissue using real-time PCR. In line with the above findings, clinical NE induced a marked increase in the accumulation of the inflammatory mRNA mediators *IFNγ*, *MMP9*, *IL23*, and *IL17* by 3.6-, 8.3-, 9.4-, and 3.1-fold, respectively, compared to the noninfected birds (Figure 6A). Notably, DCA and DCA + R 300 significantly reduced the mRNA expression of these inflammatory mediators in NE birds. Interestingly, R 300 significantly reduced Mmp9 and Il23 gene expression compared to NE.

Elevated inflammatory responses often lead to cell death. We then performed a TUNEL assay to investigate later-stage cell apoptosis because of the limited cell death antibody available for chickens. Consistent with the above analysis, clinical NE induced epithelial (blue arrow) and lamina propria immune cell death (yellow arrowhead) in the ileal villi of birds. Notably, DCA and DCA + rapamycin strongly attenuated cell death (Figure 6B), whereas rapamycin alone had lower efficacy in reducing cell death.

## 4. Discussion

*C. perfringens* remains a prevalent foodborne pathogen [32], and it is also the main pathogen of chicken NE [3]. In this study, we investigated the cellular and molecular responses to *C. perfringens*-induced NE ileitis in chickens. In particular, we dissected the role of host mTOR signaling pathways using the mTOR-specific pharmacological inhibitor rapamycin, in combination with the microbiota metabolites of the secondary bile acid DCA. Our results showed that rapamycin reduced NE-induced histopathology without attenuating the NE-induced BWG reduction, whereas DCA and DCA + rapamycin did. Rapamycin, DCA, and DCA + rapamycin restored the NE-depleted ileal total bile acid pool and reduced immune cell infiltration and ileal cell death. Together, these results suggest that mTOR mediates *C. perfringens*-induced NE ileitis and that combining DCA and rapamycin improves intervention efficacy. In this NE molecular mechanism study, we used a small number of birds, with individual birds used as an experimental unit, hence it will be important to validate the findings for poultry industry applications using a large number of birds in the future.

It is well known that overactive inflammation causes various noncommunicable gut diseases, such as inflammatory bowel disease (IBD) [33]. Although intestinal inflammation is critical to clear invading microbes and resolve infection, increasing evidence supports the notion that excessive inflammation exacerbates communicable diseases through collateral inflammation damage [34]. The hallmark of *C. perfringens*-induced NE histopathology is noted as a massive infiltration of immune cells into the intestinal lamina propria. Heterophils are important immune cells that migrate to the intestinal tissue in NE [35]. Few studies are available on the role of heterophils in inflammation, but neutrophils in mammals, as the counterpart to heterophils in chickens, have been investigated extensively. Although neutrophils engulf and destroy invading microbes, excessive infiltration of these innate immune cells causes extensive collateral tissue damage when neutrophils release various degradative enzymes and oxidative products [36]. Neutrophil infiltration into tissues causes various diseases such as IBD [37], lung injury [38], and arthritis [39]. Neutrophil accumulation causes elevated inflammatory cytokine IL1β and chemokine CXCL2 production and subsequent joint inflammation, an effect attenuated by anti-IL1β antibody treatment [40]. In the gut, phosphoinositide 3-kinases (PI3K)-mediated neutrophil infiltration promotes *C. jejuni*-induced intestinal inflammation [8]. Blocking the PI3K downstream target mTOR by rapamycin reduces neutrophil migration-mediated crypt abscesses and inflammation in mouse campylobacteriosis [12]. In this study, the inhibition of mTOR signaling by rapamycin greatly reduced NE-induced intestinal inflammation and immune cell infiltration into the intestinal lamina propria. In this report, our mechanistic study, using a cell migration assay, revealed that rapamycin reduced the infiltration of mouse immune cells into the lower chamber in the presence of *C. perfringens*. These results suggest that mTOR signaling is responsible for *C. perfringens*-induced immune cell infiltration and intestinal inflammation.

Notably, the mTOR inhibitor rapamycin, which reduced NE-induced intestinal inflammation, failed to prevent BWG loss, which is consistent with the research results showing that rapamycin reduces body weight in the biomedical field [41]. mTOR mediates a variety of homeostatic signaling pathways, such as cellular metabolism, catabolism, immune responses, autophagy, survival, proliferation, and migration [42]. Rapamycin dose-dependently reduces mouse developmental BWG and extends the life span of aged mice [43,44]. One possible mechanism of rapamycin that failed to improve BWG in this study was that it impeded NE-induced wound healing. In commercial chicken production, the parasite *Eimeria* infection is an important predisposing factor for NE [45]. To mimic NE in field chicken husbandry, birds were sequentially infected with the parasite *E. maxima* and bacterium *C. perfringens*. One consequence of parasite infection is the rupture of the intestinal line, which causes epithelial wound injury [46]. Various signaling pathways, such as the PI3K/AKT signaling pathways [47], thus activate. The PI3K downstream target mTOR is responsible for the development of multiple intestinal epithelial cell lineages and promotes stem and progenitor cell activity during intestinal epithelium repair post-injury. In a study of irradiation-induced wound injury, the epithelium-specific knockout of mTOR delayed crypt regeneration and wound healing [48]. However, in the histopathology analysis of this NE study, intestinal wounds in the rapamycin group were comparable to those in the DCA and DCA + rapamycin groups, suggesting that wound healing is not a contributing factor to rapamycin-associated inferior BWG recovery. Together, these findings suggest that rapamycin-reduced BWG possibly occurs independently of NE disease onset, which showed a slower BWG during days 23–26. In the future, it will be possible to target mTOR downstream signaling to inhibit inflammatory but not BWG pathways.

Moreover, besides DCA inhibiting *C. perfringens* virulence [49], our study showed that DCA also inhibited immune cell migration and ileal cell death. These findings are consistent with the notion that bile acids are important signaling regulators in prokaryotic and eukaryotic organisms. Research on animal molecular and cellular activities has revealed that bile acids regulate specific signaling pathways in embryogenesis, development, metabolism, and immunity [50]. Both conjugated and unconjugated bile acids activate the G-protein-coupled bile acid receptor (TGR5) with the potency in the order of LCA > DCA > CDCA > CA [51,52]. The activated TGR5 induces pathways involving cAMP, PKA, or the exchange protein directly, which is activated by cAMP (EPAC) [52,53,54]. Bile acids also activate the nuclear receptor pathways of FXR [55], pregnane X receptor (PXR) [56], and vitamin D receptor (VDR) [57]. The potency of FXR activation is CDCA > LCA > DCA > CA, whereas VDR and FXR are activated by LCA and 3-oxo-LCA [57,58]. Furthermore, bile acids mediate immune responses. Th17 cell differentiation is inhibited by 3-oxoLCA binding to the retinoid-related orphan receptor γt (RORγt), whereas isoalloLCA increases Treg cell differentiation by increasing mitochondrial reactive oxygen species (mitoROS) and FOXP3 expression [59]. IsoDCA reduces the FXR-related signaling pathways to increase Foxp3 expression [60]. Together, ours and others’ findings provide solid evidence that bile acids are signaling molecules that mediate cell and tissue homeostasis beyond the amphipathic detergent properties, which have traditionally been thought that the cellular toxicity of bile acids is mainly through non-selective detergent-like properties that disturb cellular membranes [61].

## 5. Conclusions

In summary, this study provides mechanistic insights into the role of mTOR and DCA in mediating the proinflammatory effect of *C. perfringens*-induced intestinal inflammation. mTOR- and DCA-mediated inflammation, immune cell infiltration into the intestinal lamina propria, and cell death might play an important role in the pathogenesis of NE. Intervening with *C. perfringens* infection by modulating the cellular/molecular inflammation events leading to this process could represent a new antimicrobial-free approach. Future research on the role of tissue-specific mTOR in *C. perfringens* infection could provide additional mechanistic insights.

## Data Availability

The original contributions presented in this study are included in the article. Further inquiries can be directed to the corresponding authors.
